# Genetic variants of prospectively demonstrated phenocopies in *BRCA1/2* kindreds

**DOI:** 10.1186/s13053-018-0086-0

**Published:** 2018-01-15

**Authors:** Mev Dominguez-Valentin, D. Gareth R. Evans, Sigve Nakken, Hélène Tubeuf, Daniel Vodak, Per Olaf Ekstrøm, Anke M. Nissen, Monika Morak, Elke Holinski-Feder, Alexandra Martins, Pål Møller, Eivind Hovig

**Affiliations:** 1 Department of Tumor Biology, Institute for Cancer Research, Oslo University Hospital, Oslo, Norway; 2 Department of Genetic Medicine, The University of Manchester, Manchester Academic Health Science Centre, St. Mary’s Hospital, Manchester, UK; 3 Genesis Prevention Centre, University Hospital of South Manchester, Southmoor Road, Wythenshawe, UK; 4 Inserm-U1245, UNIROUEN, Normandie Univ, Normandy Centre for Genomic and Personalized Medicine, Rouen, France; 5 Interactive Biosoftware, Rouen, France; 6 Medizinische Klinik und Poliklinik IV, Campus Innenstadt, Klinikum der Universität München, Ziemssenstr. 1, Munich, Germany; 7 MGZ—Medizinisch Genetisches Zentrum, Munich, Germany; 8 Department of Human Medicine, Universität Witten/Herdecke, Witten, Germany; 9 Department of Medical Genetics, Oslo University Hospital, Oslo, Norway; 10 Department of Informatics, University of Oslo, Oslo, Norway; 11 Institute of Cancer Genetics and Informatics, Oslo University Hospital, Oslo, Norway

**Keywords:** *BRCA1*, *BRCA2*, Breast cancer, Gene panel testing, RNA splicing

## Abstract

**Background:**

In kindreds carrying *path_BRCA1/2* variants, some women in these families will develop cancer despite testing negative for the family’s pathogenic variant. These families may have additional genetic variants, which not only may increase the susceptibility of the families’ *path_BRCA1/2,* but also be capable of causing cancer in the absence of the *path_BRCA1/2* variants. We aimed to identify novel genetic variants in prospectively detected breast cancer (BC) or gynecological cancer cases tested negative for their families’ pathogenic *BRCA1/2* variant (*path_BRCA1* or *path_BRCA2*).

**Methods:**

Women with BC or gynecological cancer who had tested negative for *path_BRCA1* or *path_BRCA2* variants were included. Forty-four cancer susceptibility genes were screened for genetic variation through a targeted amplicon-based sequencing assay. Protein- and RNA splicing-dedicated in silico analyses were performed for all variants of unknown significance (VUS). Variants predicted as the ones most likely affecting pre-mRNA splicing were experimentally analyzed in a minigene assay.

**Results:**

We identified 48 women who were tested negative for their family’s *path_BRCA1* (*n* = 13) or *path_BRCA2* (*n* = 35) variants. Pathogenic variants in the *ATM, BRCA2, MSH6* and *MUTYH* genes were found in 10% (5/48) of the cases, of whom 15% (2/13) were from *path_BRCA1* and 9% (3/35) from *path_BRCA2* families. Out of the 26 unique VUS, 3 (12%) were predicted to affect RNA splicing (*APC* c.721G > A, *MAP3K1* c.764A > G and *MSH2* c.815C > T). However, by using a minigene, assay we here show that *APC* c.721G > A does not cause a splicing defect, similarly to what has been recently reported for the *MAP3K1* c.764A > G. The *MSH2* c.815C > T was previously described as causing partial exon skipping and it was identified in this work together with the *path_BRCA2* c.9382C > T (p.R3128X).

**Conclusion:**

All women in breast or breast/ovarian cancer kindreds would benefit from being offered genetic testing irrespective of which causative genetic variants have been demonstrated in their relatives.

**Electronic supplementary material:**

The online version of this article (10.1186/s13053-018-0086-0) contains supplementary material, which is available to authorized users.

## Background

Breast cancer (BC) is one of the most common human malignancies, accounting for 22% of all cancers in women worldwide [1]. A significant proportion of BC cases can be explained by hereditary predisposition and approximately 30% of this hereditary cancer risk is explained by the currently known high-penetrance susceptibility genes [2–5]. Notably, carriers of pathogenic *BRCA1* or *BRCA2* variants (*path_BRCA1* or *path_BRCA2*) have an increased risk of developing BC (average lifetime risk of 35–85%) and ovarian cancer (average lifetime risk 11–39%). Further, carriers of pathogenic variants of *ATM, CHEK2, PALB2, NBS1* and *RAD50* have been found to confer two- to five-fold increased risk for developing BC [1, 6]. It is also known that pathogenic variants in *TP53, PTEN, STK11* and *CDH1,* resulting in Li-Fraumeni syndrome, Cowden syndrome, Peutz–Jeghers syndrome and hereditary diffuse gastric cancer, respectively, are associated with a high lifetime risk (> 40%) of BC. Moreover, pathogenic variants in *RAD51* paralogs, i.e., *RAD51C*, confer an increased risk of ovarian cancer [7]. The frequency of pathogenic variants in BC-associated genes varies significantly among different populations, as exemplified by the frequently studied founder pathogenic variant c.1100delC in *CHEK2* [6].

The identification of *path_BRCA1* or *path_BRCA2* in an affected BC individual enables access to evidence-based screening for family members, and thus facilitates the implementation of appropriate cancer prevention in these families [1, 5, 6]. However, some women in families with an identified pathogenic variant will develop cancer despite testing negative for the family’s pathogenic variant, often denoted as phenocopies [8]. In BC kindreds having a demonstrated *path_BRCA2* variant, the number of phenocopies is reportedly more frequent than expected by chance [8–10]. It has been proposed that these families may have additional genetic variants, which not only may increase the susceptibility of the families’ *path_BRCA1/2,* but also be capable of causing cancer in the absence of the *path_BRCA1/2* demonstrated in the families [5–7].

The current practice of genetic counselling for women who do not carry the *path_BRCA1/2* variants of their relatives is challenging since their recognition is crucial for application of proper diagnostic and therapeutic approaches in these families. To discover additional inherited disease-causing variants in *path_BRCA1/2* kindreds, we examined all prospectively detected BC or gynecological cancer cases in these kindreds by next-generation sequencing (NGS) using a panel of 44 cancer susceptibility genes. All detected variants were analyzed by RNA splicing- and protein-dedicated in silico methods. Variants predicted as the most likely to affect splicing were experimentally analyzed by using a cell-based minigene splicing assay.

## Methods

### Study population

For more than 20 years, we (the Hereditary Cancer Biobank from the Norwegian Radium Hospital, Norway; and the Department of Genomic Medicine from the University of Manchester, United Kingdom) have ascertained BC and breast/ovarian cancer kindreds by family history. The sisters and daughters of cancer patients were initially subjected to follow-up by annual mammography and gynecological examinations as appropriate at that time, and later they were all subjected to genetic testing [11].

Both collaborating outpatient genetic centers identified 48 women with prospective detected BC or gynecological cancer at follow-up, who were tested negative for their respective families’ *path_BRCA1/2* variants. Clinical data were obtained from pathology reports and clinical files.

Ethical approval for the prospective study was granted from the Norwegian Data Inspectorate and Ethical Review Board (ref 2015/2382). All examined patients had signed an informed consent for their participation in the study.

### Targeted sequencing

Genomic DNA was isolated from peripheral blood samples and targeted sequencing was carried out using a TrueSeq amplicon based assay v.1.5 on a MiSeq apparatus, as previously described [12]. The 44-gene panel used in this work includes genes associated with cancer predisposition as described in a prior study [12].

### Sequencing data analysis

Paired-end sequence reads were aligned to the human reference genome (build GRCh37) using the BWA-mem algorithm (v.0.7.8-r55) [13]. The initial sequence alignments were converted to BAM format and subsequently sorted and indexed with SAMtools (v.1.1) [13]. Genotyping of single nucleotide variants (SNV) and short indels was performed by GATK’s HaplotypeCaller. Filtering of raw genotype calls and assessment of callable regions/loci were done according to GATK’s best practice procedures, as described more detail previously [12].

Variants were annotated using ANNOVAR (version November 2015) [14] and were queried against a range of variant databases and protein resources (v29, December 2015), as previously described [12].

### Validation by cycling temperature capillary electrophoresis

The pathogenic variants identified in this study were validated by cycling temperature capillary electrophoresis. The method is based on allele separation by cooperative melting equilibrium while cycling the temperature surrounding capillaries [15]. This approach has previously been described and extensively used to detect somatic mutations and single nucleotide polymorphisms (SNPs) [16–19]. The amplicon design was performed by the variant melting profile tool (https://hyperbrowser.uio.no/hb/?tool_id=hb_variant_melting_profiles/) [20]. Primer sequences, PCR reaction conditions and electrophoresis settings are described in Additional file 1.

### Genetic variants nomenclature and classification

The nomenclature guidelines of the Human Genome Variation Society (HGVS) were used to describe the detected genetic variants [21]. The recurrence of the identified variants was established by interrogating six databases (in their latest releases as of November 2016): Evidence-based Network for the Interpretation of Germline Mutant Alleles (ENIGMA), Breast Cancer Information Core Database (BIC), the International Society of Gastrointestinal Hereditary Tumors (InSiGHT) Database, the Leiden Open Variation Database (LOVD), ClinVar, and the Human Gene Mutation Database (HGMD).

Novel variants were considered pathogenic if either one of the following criteria was met: a) introduced a premature stop codon in the protein sequence (nonsense or frameshift); b) occurred at positions + 1/+ 2 or − 1/− 2 of donor or acceptor splice sites, respectively; and c) represented whole-exon deletions or duplications.

### In silico analyses of VUS

Two types of bioinformatics methods were used to predict the impact of selected variants on RNA splicing. First, we used MaxEntScan (MES) and SSF-like (SSFL) to predict variant-induced alterations in 3′ and 5′ splice site strength, as described by Houdayer et al. [22], except that here both algorithms were interrogated by using the integrated software tool Alamut Batch version 1.5, (Interactive Biosoftware, http://www.interactive-biosoftware.com). For prediction of variant-induced impact on exonic splicing regulatory elements (ESR), we resorted to ΔtESRseq- [23], ΔHZei- [24], and SPANR-based [25] as described by Soukarieh et al. [26]. Score differences (Δ) between variant and wild-type (WT) cases were taken as proxies for assessing the probability of a splicing defect. More precisely, we considered that a variant mapping at a splice site was susceptible of negatively impacting exon inclusion if ΔMES≥15% and ΔSSFL≥5% [22], whereas an exonic variant located outside the splice sites was considered as a probable inducer of exon skipping if negative Δ scores (below the thresholds described below) were provided by all the 3 ESR-dedicated in silico tools. We chose the following thresholds: <− 0.5 for ΔtESRseq-, <− 10 for ΔHZei-, and < − 0.2 for SPANR-based scores. In addition, we evaluated the possibility of variant-induced de novo splice sites by taking into consideration local changes in MES and SSFL scores. In this case, we considered that variants located outside the splice sites were susceptible of creating a competing splice site if local MES scores were equal to or greater than those of the corresponding reference splice site for the same exon.

In silico protein impact predictions of VUS were performed with FATHMM (http://fathmm.biocompute.org.uk) (v2.3), PolyPhen2-HVAR (v 2.2.2), MutationTaster (data release Nov 2015), MutationAssessor (release 3), SIFT (Jan 2015) and PROVEAN (v1.1 Jan 2015) using dbNSFP v3.4.

### Cell-based minigene splicing assays

In order to determine the impact of the *APC* c.721G > A on RNA splicing, we performed functional assays based on the comparative analysis of the splicing pattern of WT and mutant reporter minigenes [27], as follows. First, the genomic region containing *APC* exon 7 and at least 150 nucleotides of the flanking introns (c.646–169 to c.729 + 247) were amplified by PCR using patient #12470 DNA as template and primers indicated in Additional file 2. Next, the PCR-amplified fragments were inserted into a previously linearized pCAS2 vector [26] to generate the pCAS2-APC exon 7 WT and c.721G > A minigenes. All constructs were sequenced to ensure that no unwanted mutations had been introduced into the inserted fragments during PCR or cloning. Then, WT and mutant minigenes were transfected in parallel into HeLa cells grown in 12-well plates (at ~ 70% confluence) using the FuGENE 6 transfection reagent (Roche Applied Science). Twenty-four hours later, total RNA was extracted using the NucleoSpin RNA II kit (Macherey Nagel) and, the minigene transcripts were analyzed by semi-quantitative RT-PCR using the OneStep RT-PCR kit (QIAGEN), as previously described [26]. The sequences of the RT-PCR primers are shown in Additional file 2. Then, RT-PCR products were separated by electrophoresis on 2.5% agarose gel containing EtBr and visualized by exposure to UV light under saturating conditions using the Gel Doc XR image acquisition system (Bio-Rad), followed by gel-purification and Sanger sequencing for proper identification of the minigenes’ transcripts. Finally, splicing events were quantitated by performing equivalent fluorescent RT-PCR reactions followed by capillary electrophoresis on an automated sequencer (Applied Biosystems), and computational analysis by using the GeneMapper v5.0 software (Applied Biosystems).

## Results

### Family history and clinical characteristics

**Fig. 1:**
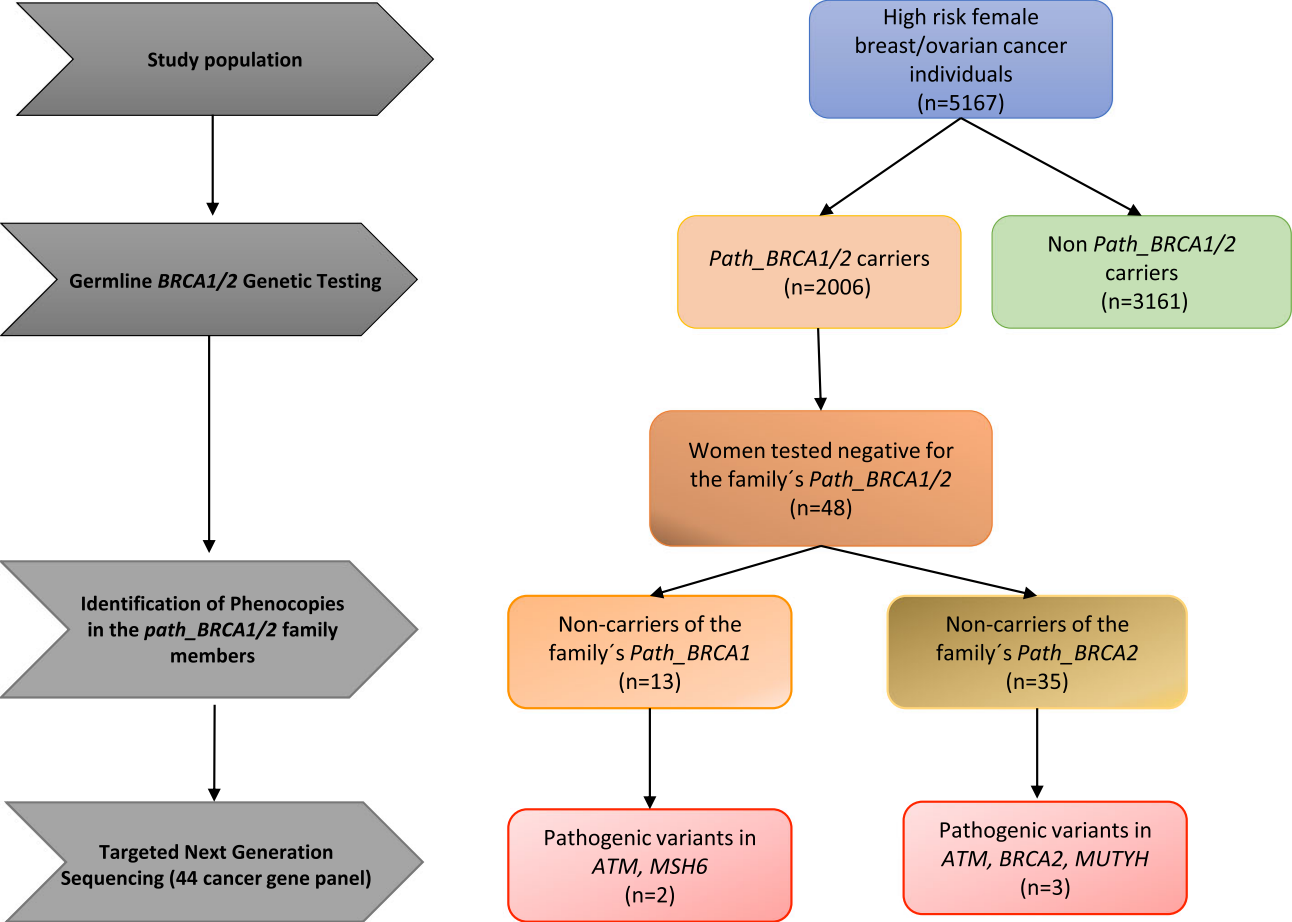
Flow chart showing the study population selection from the Hereditary Cancer Biobank from the Norwegian Radium Hospital, Norway. It contains ascertained BC and breast/ovarian cancer kindreds by family history that were all subjected to genetic testing. The identification of phenocopies involved 48 women with prospective detected BC or gynecological cancer at follow-up, who were tested negative for their respective families’ *path_BRCA1/2* variants. Among these cases, 13 were identified in non-carriers of the family’s *path_BRCA1* variant and in 35 non-carriers of the family’s *path_BRCA2* variant (*n* = 30 from the Department of Genomic Medicine from the University of Manchester). Pathogenic variants were identified in 5/48 (10%) BC or gynecological cancer cases

In total, we identified 48 cases, of whom 18 BC or gynecological cancer patients who did not carry their respective families’ *path_BRCA1* or *path_BRCA2* variants (*n* = 13 and *n* = 5, respectively) came from the Hereditary Cancer Biobank from the Norwegian Radium Hospital, while the Department of Genomic Medicine from the University of Manchester identified a total of 30 BC patients, all non-carriers of the family’s *path_BRCA2* variants (Fig. 1)*.* The median age at first cancer diagnosis was 53.5 years (range 31–79 years). The incidence was higher for BC (92%), followed by ovarian cancer (4%) and endometrial and cervical cancer (2% each) (Table 1).

**Table 1:** Summary of the 48 prospective BC or gynecological cancer patients included in the study

Patient_ID	Institution	Familial path_BRCA1 or path_BRCA2 variantFamilial path_BRCA1 or path_BRCA2 variant	ICD9 diagnosis (age)	Pathogenic variant identified in the current study
17,161	HCBNRH	*BRCA2 c.5217_5223delTTTAAGT (p.Tyr1739Terfs)BRCA2 c.5217_5223delTTTAAGT (p.Tyr1739Terfs)*	OC (67)	ATM c.468G > A (p.Trp156Ter)*ATM c.468G > A (p.Trp156Ter)*
6475	HCBNRH	*BRCA1 c.1011dupA (p.Val340Glyfs)BRCA1 c.1011dupA (p.Val340Glyfs)*	BC (52)	*ATM c.9139C > T (p.Arg3047Ter)ATM c.9139C > T (p.Arg3047Ter)*
13,141	HCBNRH	*BRCA1 c.1072delC (p.Leu358Cysfs)BRCA1 c.1072delC (p.Leu358Cysfs)*	EC (57)	MSH6 c.2864delC (p.Thr955fs)*MSH6 c.2864delC (p.Thr955fs)*
1873	HCBNRH	*BRCA1 c.1556delA (p.Lys519Argfs)BRCA1 c.1556delA (p.Lys519Argfs)*	MTHM (56), BC (70)	Not
5378	HCBNRH	*BRCA1 c.697_698delGT (p.Val233Asnfs)BRCA1 c.697_698delGT (p.Val233Asnfs)*	BC (52)	Not
5180	HCBNRH	*BRCA1 c.5194-2A > CBRCA1 c.5194-2A > C*	BC (39)	Not
22	HCBNRH	*BRCA2 c.3847_3848delGT (p.Val1283Lysfs)BRCA2 c.3847_3848delGT (p.Val1283Lysfs)*	BC (63)	Not
243	HCBNRH	*BRCA2 c.3847_3848delGT (p.Val1283Lysfs)BRCA2 c.3847_3848delGT (p.Val1283Lysfs)*	CVC (41)	Not
5348	HCBNRH	*BRCA1 c.1556delA (p.Lys519Argfs)BRCA1 c.1556delA (p.Lys519Argfs)*	BC (68)	Not
6031	HCBNRH	*BRCA1 c.1556delA (p.Lys519Argfs)BRCA1 c.1556delA (p.Lys519Argfs)*	BC (66)	Not
6032	HCBNRH	*BRCA1 c.3228_3229delAG (p.Gly1077Alafs)BRCA1 c.3228_3229delAG (p.Gly1077Alafs)*	OC (55)	Not
6207	HCBNRH	*BRCA1 c.697_698delGT (p.Val233Asnfs)BRCA1 c.697_698delGT (p.Val233Asnfs)*	BC (47)	Not
8085	HCBNRH	BRCA1 c.3228_3229delAG (p.Gly1077Alafs)BRCA1 c.3228_3229delAG (p.Gly1077Alafs)	BC (55), CC (66)	Not
11,717	HCBNRH	BRCA1 c.1556delA (p.Lys519Argfs)BRCA1 c.1556delA (p.Lys519Argfs)	BC(42,57)	Not
12,470	HCBNRH	*BRCA1 c.3178G > T (p.Glu1060Ter)*	BC (39)	Not
13,023	HCBNRH	*BRCA2 c.5217_5223delTTTAAGT (p.Tyr1739Terfs)*	BC (59)	Not
15,529	HCBNRH	*BRCA2 c.4821_4823delTGAins*	BC (48)	Not
22,325	HCBNRH	*BRCA1 c.5047G > T (p.Glu1683Ter)*	BC (45)	Not
1,100,948	UM	*BRCA2 c.6591_6592delTG (p.Glu2198Asnfs)*	BC (44)	*BRCA2 c.9382C > T (p.Arg3128Ter)*
12,010,643	UM	*BRCA2 c.7360delA (p.Ile2454Phefs)*	BC (56)	*MUTYH c.1178G > A (p.Gly393Asp)*
75,443	UM	*BRCA2 c.5909C > A (p.Ser1970Ter)*	BC (55)	Not
88,295	UM	*BRCA2 c.7977-1G > C*	BC (44)	Not
64,949	UM	*BRCA2 c.5909C > A (p.Ser1970Ter)*	BC (55)	Not
67,723	UM	BRCA2 c.4866delA p.(Arg1622Serfs*14)	BC (46)	Not
84,510	UM	*BRCA2 c.5946delT (p.Ser1982Argfs)*	BC (67)	Not
13,007,862	UM	BRCA2 c.5909C > A (p.Ser1970Ter)	BC (31)	Not
9,009,462	UM	BRCA2 c.6535_6536insA (p.Val2179Aspfs)	BC (67)	Not
900,178	UM	BRCA2 c.1889delC (p.Thr630Asnfs)	BC (49,77)	Not
10,005,829	UM	*BRCA2 c.9541_9554del p.(Met318CysfsTer13)*	BC (38)	Not
10,007,016	UM	BRCA2 c.632-1G > A	BC (51)	Not
10,003,959	UM	*BRCA2 c.6275_6276delTT (p.Leu2092Profs)*	BC (55)	Not
12,852	UM	*BRCA2 c.1929delG (p.Arg645Glufs)*	BC (56)	Not
12,001,161	UM	BRCA2 c.7958 T > C (p.Leu2653Pro)	BC (67)	Not
13,017,067	UM	BRCA2 c.755_758delACAG (p.Asp252Valfs)	BC (74)	Not
688	UM	BRCA2 c.1929delG (p.Arg645Glufs)	BC (32)	Not
40,540	UM	BRCA2 c.8535_8538delAGAG p.(Glu2846LysfsTer16)	BC (69)	Not
9,001,644	UM	BRCA2 c.4965C > G (p.Tyr1655Ter)	BC (39, 45)	Not
89,205	UM	BRCA2 c.5946delT (p.Ser1982Argfs)	BC (77)	Not
10,002,068	UM	BRCA2 del exons 14–16	BC (37)	Not
10,004,590	UM	BRCA2 c.2672dupT	BC (67,67)	Not
40,286	UM	BRCA2 c.7069_7070delCT p.(Leu2357ValfsTer2)	BC (36,53)	Not
76,618	UM	BRCA2 c.4478_4481delAAAG (p.Glu1493Valfs)	BC (51)	Not
12,015,576	UM	BRCA2 c.9382C > T (p.Arg3128Ter)	BC (45)	Not
61,420	UM	BRCA2 c.5350_5351delAA p.(Asn1784HisfsTer2)	BC (59)	Not
960,579	UM	BRCA2 c.2808_2811del4 (p.Ala938Profs)	BC (39)	Not
14,965	UM	BRCA2 c.5682C > G p.(Tyr1894Ter)	BC (59)	Not
20,468	UM	*BRCA2 c.6275_6276delTT (p.Leu2092Profs)*	BC (38)	Not
56,193	UM	*BRCA2 c.7884dupA (p.Trp2629Metfs)*	BC (79)	Not

*HCBNRH* Hereditary Cancer Biobank from the Norwegian Radium Hospital (Norway), *UM* University of Manchester (United Kingdom), *ICD9 diagnosis* International Classification of Diseases, 9th Revision, *OC* Ovary cancer, *BC* Breast cancer, *EC* Endometrial cancer, *MTHM* Malignant neoplasm of thymus, heart, and mediastinum, *CC* Colon cancer, *CVC* Cervical cancer, *Considered pathogenic based in its nature (nonsense and frameshift), *VUS* Variants of unknown significance, *NM for ATM* NM_000051, *BRCA1* NM_007294.3, *BRCA2* NM_000059.3, *MSH6* NM_001281492, *MUTYH* NM_012222

### Germline findings

In the 48 cases, we identified five (10%) to carry pathogenic variants in *ATM* (c.468G > A, p.Trp156Ter and c.9139C > T, p.Arg3047Ter)*, BRCA2* (c.9382C > T, p.Arg3128Ter)*, MSH6* (c.2864delC, p.Thr955fs) and *MUTYH* (c.1178G > A, p.Gly393Asp). Among these five cases, 2/13 were identified in non-carriers of the family’s *path_BRCA1* variant and in 3/35 non-carriers of the family’s *path_BRCA2* variant (Fig. 1). Disease type, familial *path_BRCA1/2* and pathogenic variants found in this study are shown in detail in Table 1.

Interestingly, one case with a familial *path_BRCA2* **(**c.6591_6592delTG) was found to carry another pathogenic variant in the same gene (*BRCA2* c.9382C > T, p.Arg3128Ter), which causes a premature stop in the codon 3128 and is known to be a high risk pathogenic variant (Table 1).

The pathogenic variants in BC-related genes (2 in *ATM* and 1 in *BRCA2*) were found in 3 women with BC or ovarian cancer, while the *MSH6* and the heterozygous *MUTYH* p.Gly393Asp pathogenic variant was found in a woman with endometrial cancer at 57 years and BC diagnosis at 56 years, respectively (Table 1).

### Validation of the cancer gene panel output

The presence of the five pathogenic variants detected by targeted NGS was confirmed by cycling temperature capillary electrophoresis, showing 100% correspondence between both methods.

### Variants of unknown significance (VUS) and predicted protein alterations

In total, we found 26 unique VUS in 30 out of 48 patients (63%). Common polymorphisms (with an allele frequency ≥ 1% in the general population according to the ExAC database) and benign variants classified according to either ClinVar or the American College of Medical Genetics and Genomics (ACMG) guidelines were excluded from further analyses [41, 58].

The VUS were detected in 17 genes, namely: *AXIN2*, *RAD51B* (in 4 patients each), *MAP3K1* (in 3 patients), *APC*, *ATM*, *MSH2, NBN, POLE* (in 2 patients each), *BRCA1*, *CDH1*, *CDX2*, *DVL2*, *MRE11A*, *MUTYH*, *NOTCH3*, *PTEN* and *RAD51D* (in 1 patient each) (Table 2). The minor allele frequencies (MAF) of these variants in public databases were very low or no frequency data have been reported (Table 2).

**Table 2:** RNA splicing- dedicated in silico analyses for the VUS identified in our study

Patient ID	Genomic position (GRCh37)	Gene	Exon	Nucleotide change (cNomen)	Predicted protein change (pNomen)	dbSNPrsID	Non-Finnish European population frequency*	Reference splice site-dedicated analyses								Cryptic splice site-dedicated analyses			ESR-dedicated analyses		
								Nearest reference		MES scores			SSFL scores			Potential local splice effect	Local MES scores		∆tESRseq	∆Hzei	ΔΨ
								Distance	Type	WT	Var	VAR vs WT	WT	Var	VAR vs WT		WT	Var			
								(nt)	(3′ or 5’ss)			∆ (%)			∆ (%)						
688	chr_16_68835593_G_A	*CDH1*	3	c.184G > A	p.Gly62Ser	587,781,898	5.99e-05	21	3’	8.17477	8.17477	0	86.5179	86.5179	0				− 1.44947	10.35	−1.24
	chr2_47703664_G_A	*MSH2*	13	c.2164G > A	p.Val722Ile	587,781,996	8.99e-05	−47	5’	10.8583	10.8583	0	100	100	0				0.59756	10.51	−0.01
	chr_8_90983475_C_A	*NBN*	6	c.628G > T	p.Val210Phe	61,754,796	0.0008158	44	3’	6.19815	6.19815	0	86.8244	86.8244	0				− 0.782222	−46.21	− 0.15
1873	chr_5_56155672_A_G	*MAP3K1*	3	c.764A > G	p.Asn255Ser	56,069,227	0.0269	−71	5’	7.52484	7.52484	0	78.4708	78.4708	0	New Acceptor Site?	–	8.8	−1.18661	6.7	−0.04
5378	chr 12_133244944_G_A	*POLE*	19	c.2171C > T	p.Ala724Val	61,734,163	0.00030	−3	5’	9.89081	8.73118	−11.7	86.6769	82.5488	−4.8	New Donor Site?	–	6.3	−2.14822	−32.05	−0.16
6031	chr17_41245621_T_C	*BRCA1*	10	c.1927A > G	p.Ser643Gly	80,357,105	NA	1257	3’	8.86265	8.86265	0	87.3058	87.3058	0				1.44078	58.08	0.02
		*AXIN2*	10	c.2272G > A	p.Ala758Thr	145,007,501	0.0039861	35	3’	6.34671	6.34671	0	86.1925	86.1925	0				−0.942617	0.12	−0.09
	chr5_112102960_C_T	*APC*	4	c.295C > T	p.Arg99Trp	139,196,838	0.0006444	75	3’	7.49577	7.49577	0	84.8039	84.8039	0				−2.2189	−14.34	− 0.08
12,470		*AXIN2*	10	c.2272G > A	p.Ala758Thr	145,007,501	0.0039861	35	3’	6.34671	6.34671	0	86.1925	86.1925	0				−0.942617	0.12	− 0.09
	chr5_112128218_G_A	*APC*	7	c.721G > A	p.Glu241Lys	777,603,154	0.0001818	−9	5’	7.15277	7.15277		87.0697	87.0697	0				−1.51981	−49.76	−0.42
12,852	chr_14_69061228_G_A	*RAD51B RAD51B*	11	c.1063G > A	p.Ala355Thr	61,758,785	0.0071658	27	3’	11.8	11.8	0	80.2	80.2	0	–	–	–	−1.24035	−50.64	–
88,295	chr10_89690828_G_A	*PTEN PTEN*	4	c.235G > A	p.Ala79Thr	202,004,587	0.0001678	−19	5’	9.6515	9.6515	0	86.8647	86.8647	0				−1.39321	10.77	0.6
900,178	chr11_94197365_C_T	*MRE11A MRE11A*	11	c.1139G > A	p.Arg380His	587,781,646	4.5e-05	41	3’	8.9941	8.9941	0	95.7456	95.7456	0				−1.57887	−48.78	−0.03
960,579	chr_5_56177843_C_G	*MAP3K1*	14	c.2816C > G	p.Ser939Cys	45,556,841	0.0221	447	3’	12.0063	12.0063	0	100	100	0	–	–	–	−0.486881	−16.1	0
1,000,459	chr13_28537449_ACTT_A	*CDX2*	3	c.742_744del	p.Lys248delAAG	553,066,746	0.0001682	55	3’	11.7045	11.7045	0	87.4307	87.4307	0				−2.46964	−100.08	–
1,100,948	chr_17_7133187_A_G	*DVL2 DVL2*	5	c.596 T > C	p.Met199Thr	372,715,697	6.01e-05	−61	5’	6.34467	6.34467	0	80.4452	80.4452	0				0.0509416	−1.77	0.54
	chr2_47641430_C_T	*MSH2*	5	c.815C > T	p.Ala272Val	34,136,999	0.0003755	23	3’	10.3527	10.3527	0	84.3224	84.3224	0				−2.17832	−46.5	−0.03
10,002,068	chr_17_63526198_C_T	*AXIN2*	11	c.2428G > A	p.Asp810Asn	140,344,858	1.5e-05	23	3’	11.6727	11.6727	0	87.3948	87.3948	0				−1.22987	−14.33	–
10,005,829	chr_14_69061228_G_A	*RAD51B*	11	c.1063G > A	p.Ala355Thr	61,758,785	0.0071658	27	3’	11.8	11.8	0	80.2	80.2	0	–	–	–	−1.24035	−50.64	–
	chr8_90993640_C_T	*NBN*	3	c.283G > A	p.Asp95Asn	61,753,720	0.0030459	−38	5’	10.7663	10.7663	0	94.6711	94.6711	0				0.318238	24.6	0.03
	chr_11_108155132_G_A	*ATM*	26	c.3925G > A	p.Ala1309Thr	149,711,770	0.0009147			9.98517	9.98517	0	84.8076	84.8076	0				0.676556	32.96	0.04
12,001,161	chr_14_68353893_A_G	*RAD51B*	7	c.728A > G	p.Lys243Arg	34,594,234	0.010682	−29	5’	9.09184	9.09184	0	78.9497	78.9497	0	Cryptic 5’ss activation?	0.9	7.9	−1.48785	−40.54	−0.19
12,015,576	chr19_15291551_C_G	*NOTCH3*	19	c.3083G > C	p.Trp1028Ser	rs146829488	na	−60	5’	11.1124	11.1124	0	82.5954	82.5954	0				0.300115	−6.4	0.1
11,717	chr1_45797881_C_T	*MUTYH*	10	c.881G > A	p.Cys294Tyr	rs879254257	na	−44	5’	6.31089	6.31089	0	72.818	72.818	0				1.09496	−7.06	0.04
17,161	chr_11_108139187_T_A	*ATM*	18	c.2689 T > A	p.Phe897Ile	147,122,522	4.5e-05	51	3’	9.8979	9.8979	0	93.4253	93.4253	0				0.554269	87.95	0.01
22	chr_12_133241897_A_G	*POLE*	21	c.2459 T > C	p.Met820Thr	767,460,640	0	−10	5’	6.58677	6.58677	0	77.9039	77.9039	0				1.28743	−2.13	0.06
	chr_14_68352672_A_G	*RAD51B*	6	c.539A > G	p.Tyr180Cys	28,910,275	0.0045906	−34	5’	9.54919	9.54919	0	83.7411	83.7411	0				0.881539	7.23	−0.19
	chr_5_56155672_A_G	*MAP3K1*	3	c.764A > G	p.Asn255Ser	56,069,227	0.0269	−71	5’	7.52484	7.52484	0	78.4708	78.4708	0	New Acceptor Site?	–	8.8	−1.18661	6.7	−0.04
6207	chr_17_63530163_C_T	*AXIN2*	10	c.2272G > A	p.Ala758Thr	145,007,501	0.0039861	35	3’	6.34671	6.34671	0	86.1925	86.1925	0				−0.942617	0.12	−0.09
6475	chr_17_33433488_G_A	*RAD51D*	6	c.493C > T	p.Arg165Trp	544,654,228	6.94e-05	13	3’	8.20686	8.20686	0	85.1161	85.1161	0				−2.55724	−22.32	1.42

*na* not available; *Non-Finnish European population based on ExAC database; NM for APC: NM_000038; ATM: NM_000051; AXIN2: NM_004655; BRCA1: NM_007300; CDH1: NM_004360; CDX2: NM_001265; DVL2: NM_004422; MAP3K1: NM_005921; MSH2: NM_000251; MRE11A: NM_005591; MUTYH: NM_012222; NBN: NM_002485; NOTCH3: NM_000435; POLE: NM_006231; PTEN: NM_000314; RAD51B: NM_133509; RAD51D: NM_002878. In order to predict their biological impact, RNA splicing-dedicated bioinformatics analyses were performed as described under Materials and Methods. Results shown in bold were considered as predictive of a potential variant-induced negative biological effect. *MES* MaxEntScan, *SSFL* Splice Site Finder-Like, *nt* Nucleotide, *3′ or 5’ss* 3′ splice site or 5′ splice site, *ESR* Exonic splicing regulators

**Fig. 2:**
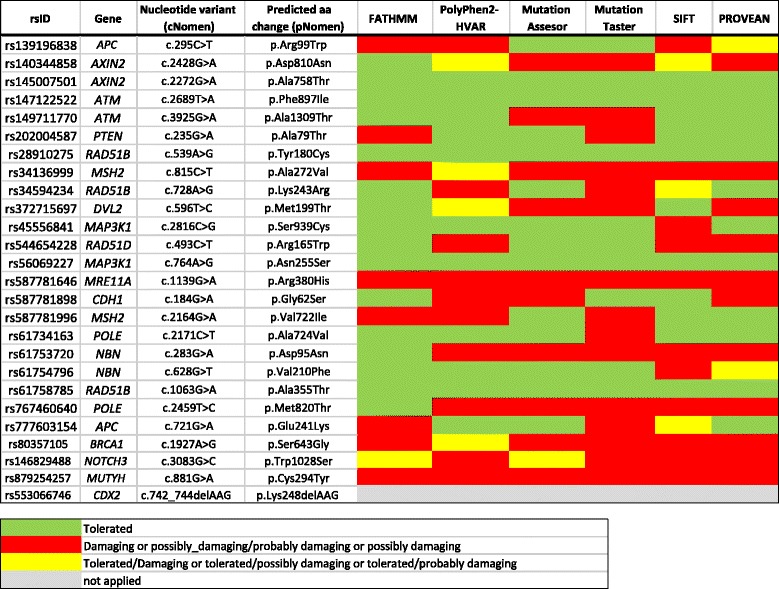
Protein-related in silico data obtained for the VUS identified in the study

The VUS were furthermore analyzed by using 6 in silico protein prediction tools with different underlying algorithms (Fig. 2). The *MRE11A* c.1139G > A and the *MUTYH* c.881G > A variants were suggested to have a potentially damaging effect on protein level by all six predictions programs. For the variants in the *MSH2, NBN, POLE* and *BRCA1* genes (*MSH2* c.815C > T, *NBN* c.283G > A, *POLE* c.2459 T > C and *BRCA1* c.1927A > G, five out of six predictions suggested a potentially damaging effect (Fig. 2).

Discrepancies in protein-related predictions were even more pronounced for the variants in *APC*, *AXIN2, RAD51B, DVL2, RAD51D, CDH1* and *MSH2* c.2164G > A. In contrast, none of the six prediction tools showed deleterious effects for the detected variants in the *AXIN2, ATM, RAD51B* and *MAP3K1* genes (*AXIN2* c.2272G > A, *ATM* c.2689 T > A, *RAD51B* c.539A > G and c.1063G > A and *MAP3K1* c.764A > G) (Fig. 2).

### *Splicing-dedicated* in silico *analysis and minigene splicing assay*s

**Fig. 3:**
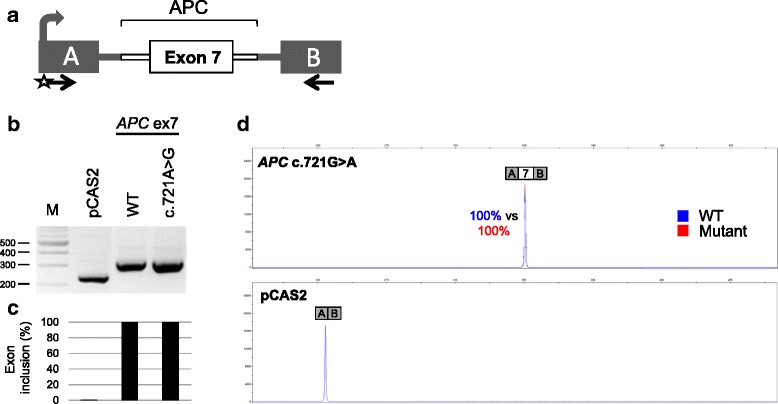
Analysis of the impact on RNA splicing of *APC* c.721G > A by using a cell-based minigene splicing assay. **a** Structure of pCAS2-APC.ex7 minigene used in the assay. The bent arrow indicates the CMV promoter, boxes represent exons, lines in between indicate introns, and arrows below the exons represent primers used in RT-PCR reactions. The WT and c.721G > A minigenes were generated by inserting a genomic fragment containing the exon of interest and flanking intronic sequences into the intron of pCAS2, as described under Materials and Methods. **b** Analysis of the splicing pattern of pCAS2-APC.ex7 WT and c.721G > A minigenes. The two constructs were introduced into HeLa cells and the minigenes’ transcripts were analyzed by RT-PCR 24 h post-transfection. The image shows the results of a representative experiment in which the RT-PCR products were separated on a 2.5% agarose gel stained with EtBr and visualized by exposure to ultraviolet light. M, 100 bp DNA ladder (New England Biolabs). **c** Quantification of splicing events observed in the minigene splicing assay. The relative levels of exon inclusion indicated under the gel are based on RT-PCR experiments equivalent to those shown in B but performed with a fluorescent forward primer and then separated on an automated sequencer under denaturing conditions. Quantification results were obtained by using the GeneMapper v5.0 software (Applied Biosystems) and correspond to the average of two independent fluorescent-RT-PCR experiments. **d** Representative fluorescent RT-PCR experiment. The panel shows superposed peaks corresponding to the WT and mutant products (in blue and red, respectively), as indicated

Out of the 26 unique VUS, two (*APC* c.721G > A and *MAP3K1* c.764A > G) were bioinformatically predicted as the most likely to affect RNA splicing, either by potentially creating a new splice site or by altering putative exonic splicing regulatory elements, respectively (Table 2). Given that RNA data was not available for *APC* c.721G > A, we set out to experimentally evaluate the impact on RNA splicing produced by this variant, by performing a cell-based minigene splicing assay. As shown in Fig. 3, we observed that c.721G > A did not affect the splicing pattern of *APC* exon 7 in our system. These results are reminiscent of those recently obtained for *MAP3K1* c.764A > G by using a similar splicing assay, in which the variant did not cause an alteration in the minigene’s splicing pattern (Dominguez-Valentin et al. *under submission*). It would be important in both cases to validate the minigene results by analyzing RNA from the variant carriers/patients as compared to those from healthy controls. However, we do not have such material in our biobank.

To our knowledge, the only other VUS from our list for which RNA data is available is *MSH2* c.815C > T (p.Ala272Val). Previous results from different minigene assays revealed that, albeit located outside the splice sites, *MSH2* c.815C > T induces partial skipping of exon 5 [28]. These results agree, at least in part, with those obtained by analyzing RNA from a LS patient carrying this same variant [29]. Indeed, the latter study revealed aberrantly spliced *MSH2* transcripts associated with the presence of c.815C > T, but where the severity of the splicing defect was not addressed at the time. Of note, here we identified *MSH2* c.815C > T together with another VUS (*DVL2* c.596 T > C) and a *path_BRCA2* c.9382C > T (different from the familial *path_BRCA2*) in a patient diagnosed with ductal carcinoma at 44 years of age (Patient 1,100,948) (Table 1).

## Discussion

Among prospectively detected BC or gynecological cancer phenocopies in the *path_BRCA1/2* families, we found that 4/48 have pathogenic variants in high-penetrance cancer genes: two BC- and one CRC-associated gene (*ATM, BRCA2* and *MSH6,* respectively). Our findings are in line with a previous study, which detected a likely pathogenic variant in a gene other than *BRCA1/2* in a BC patient, i.e. *MSH6* c.3848_3862del (p.(Ile1283_Tyr1287del) [30]. In addition, we found the *MUTYH* c.1178G > A (p.Gly393Asp) variant in a BC case, which is one of the most common *path*_*MUTYH* variants. Pathogenic *MUTYH* variants may cause a recessively inherited colon cancer syndrome. Whether or not individuals who are heterozygous for *MUTYH* mutations may be at risk for cancer is debated [31]. Among the five cases found to carry pathogenic variants, 2/13 were identified from families with *path_BRCA1* and 3/35 with *path_BRCA2* variants.

Our results are in concordance with the recently published NGS panel studies, which have demonstrated that besides high-risk genes, like *BRCA1*/*2* and MMR genes, other genes may also contribute to familial cancer predisposition, thus providing a broader picture on the genetic heterogeneity of cancer syndromes [25, 32, 33]. In this regard, a molecular diagnosis yield of approximately 9% to identify a pathogenic or likely pathogenic variant in BC has been reported, and with yields of 13% in ovarian and 15% in colon/stomach cancer cases [25]. On the other hand, family history is currently used to identify high risk patients. However, the use of family history fails to identify women without close female relatives who are carriers of pathogenic variants [9].

Despite the potential of NGS to identify genetic causes among families that tested negative for pathogenic variants in high-risk genes using traditional methods [25, 32, 33], a high number of VUS are also detected and constitute a major challenge in oncogenetics [34]. In this study, we subjected 26 VUS to RNA splicing and protein in silico evaluations, and the bioinformatics predictions indicated that two VUS (*APC* c.721G > A and *MAP3K1* c.764A > G) were likely to affect RNA splicing. Our results from minigene splicing assays suggest, however, that this is not the case. Complementary analysis of patients’ RNA will be important to verify the impact on splicing of these variants in vivo. Of note, none of the six protein in silico prediction tools showed a deleterious effect for the *MAP3K1* c.764A > G missense variant and inconsistences were found for the *APC* c.721G > A variant.

Bioinformatics prediction tools are widely used to aid the biological and clinical interpretation of sequence variants, although it is well recognized that they have their limitations. Co-segregation studies for further evaluation will be key for understanding whether some of the VUS detected in this work may have a causal effect. Some of the VUS may in the future be reclassified as deleterious or benign, but in the meantime, they cannot be used to make clinical decisions [30].

A polygenic model involving a combination of multiple genomic risk factors, including the effect of low- or moderate- penetrance susceptibility alleles may explain the increased BC risk in women who tested negative for family’s *path_BRCA1/2* variants [5]. In addition, heterozygous whole gene deletions (WGD) and intragenic microdeletions have been reported to account for a significant proportion of pathogenic variants underlying cancer predisposition syndromes, although WGD were not a common mechanism in any of the three high-risk BC genes, *BRCA1, BRCA2* and *TP53* [35].

The clinical utility of gene panels such as the one used in this study is not yet fully established and the appropriate routes for clinical deployment of such tests remain under discussion [36]. So far, the large patient datasets generated by NGS panels may be used to explore the specific penetrance of the genes included in these panels, and to assess the performance and implications of the use of NGS in clinical diagnostics [34].

## Conclusions

In kindreds carrying *path_BRCA1/2* variants, testing only for the already known *path_BRCA1/2* variants in the family may not be sufficient to exclude increased risk neither for BC nor for ovarian cancer or other cancers in the healthy female relatives. Our findings suggest that all women in BC or breast/ovarian cancer kindreds would benefit from being offered genetic testing irrespective of which causative genetic variants have been demonstrated in their relatives*.* In addition, we found a number of VUS in genes other than *BRCA1/2* i.e. *AXIN2, APC, DVL2*, *MAP3K1*, *RAD51B, NBN, POLE*, *CDH1*, *CDX2*, *MRE11A*, *MUTYH*, *NOTCH3, PTEN* and *RAD51D.* All these may be suspected of being associated with cancer in the families studied and may be considered as candidates for being included in future gene panel testing to better understand why some families present aggregation of cancer cases.

## Additional files

Additional file 1: The concentration in a 10 ml PCR was 1xThermopol Reaction Buffer with 2 mM MgS04, 0.3 μM “reverse” primers, 0.15 μM “forward” primer, 0.1 μM, 6-Carboxyfluorescein-GC clamp primer, 600 μM dNTP, 100 μg Bovine Serum Albumine (Sigma-Aldrich, Oslo, Norway) and 0.75 U Taq DNA polymerase. Plates were sealed with two strips of electrical tape (Clas Ohlson, Oslo, Norway). The temperature cycling was repeated 35 times; 94 °C for 30 s, annealing temperature held for 30 s and extension at 72 °C for 60 s (Eppendorf Mastercycler ep gradient S (Eppendorf, Hamburg, Germany)). **Table S1.** primers used to amplify PCR product to be analysed by cycling temperature capillary electrophoresis. (DOCX 16 kb)

Additional file 2: Primers used in the pCAS2 minigene splicing assay. (DOCX 14 kb)

## Abbreviations

ACMG: American College of Medical Genetics and Genomics
BC: Breast cancer
BIC: Breast Cancer Information Core Database
CRC: Colorectal cancer
ENIGMA: Evidence-based Network for the Interpretation of Germline Mutant Alleles
ESR: Exonic splicing regulatory elements
HGMD: Human Gene Mutation Database
InSiGHT: International Society of Gastrointestinal Hereditary Tumors Database
LOVD: Leiden Open Variation Database
LS: Lynch syndrome
MAF: Minor allele frequency
MES: MaxEntScan
NGS: Next generation sequencing
*path_BRCA1/2*: Pathogenic (disease-causing) variant of the *BRCA*1 or the *BRCA2* genes
SNPs: Single nucleotide polymorphisms
SNV: Single-nucleotide variants
SSFL: SSF-like
VUS: Variants of unknown significance
WGD: Whole gene deletions
WT: Wild type
