# Supplementary material for: Genetic variants of prospectively demonstrated phenocopies in BRCA1/2 kindreds
Source: Hered Cancer Clin Pract. 2018 Jan 15;16:4. doi: 10.1186/s13053-018-0086-0 (PMC5769521; doi:10.1186/s13053-018-0086-0)
Supplement: Supplementary file 1 — The concentration in a 10 ml PCR was 1xThermopol Reaction Buffer with 2 mM MgS04, 0.3 μM “reverse” primers, 0.15 μM “forward” primer, 0.1 μM, 6-Carboxyfluorescein-GC clamp primer, 600 μM dNTP, 100 μg Bovine Serum Albumine (Sigma-Aldrich, Oslo, Norway) and 0.75 U Taq DNA polymerase. Plates were sealed with two strips of electrical tape (Clas Ohlson, Oslo, Norway). The temperature cycling was repeated 35 times; 94 °C for 30 s, annealing temperature held for 30 s and extension at 72 °C for 60 s (Eppendorf Mastercycler ep gradient S (Eppendorf, Hamburg, Germany)). Table S1. primers used to amplify PCR product to be analysed by cycling temperature capillary electrophoresis. (DOCX 16 kb) [file 13053_2018_86_MOESM1_ESM.docx]

**Supporting information 1.**

The concentration in a 10 ml PCR was 1xThermopol Reaction Buffer with 2 mM MgS04, 0.3 μM “reverse” primers, 0.15 μM “forward” primer, 0.1 μM, 6-Carboxyfluorescein-GC clamp primer, 600 μM dNTP, 100 μg Bovine Serum Albumine (Sigma-Aldrich, Oslo, Norway) and 0.75 U Taq DNA polymerase. Plates were sealed with two strips of electrical tape (Clas Ohlson, Oslo, Norway). The temperature cycling was repeated 35 times; 94 °C for 30 s, annealing temperature held for 30 s and extension at 72 °C for 60 s (Eppendorf Mastercycler ep gradient S (Eppendorf, Hamburg, Germany)).

**Supporting Table 1**. Primers used to amplify PCR product to be analysed by cycling temperature capillary electrophoresis.

Melting profile of the fragment generated by

<https://hyperbrowser.uio.no/hb/tool_runner/data_source_redirect?tool_id=hb_variant_melting_profiles>
