# Supplementary material for: Genetic variants of prospectively demonstrated phenocopies in BRCA1/2 kindreds
Source: Hered Cancer Clin Pract. 2018 Jan 15;16:4. doi: 10.1186/s13053-018-0086-0 (PMC5769521; doi:10.1186/s13053-018-0086-0)
Supplement: Supplementary file 2 — Primers used in the pCAS2 minigene splicing assay. (DOCX 14 kb) [file 13053_2018_86_MOESM2_ESM.docx]

| Purpose | Forward (F) or reverse (R) primers | |
| --- | --- | --- |
|  | Name | Sequence (5’-3’) |
| PCR (cloning, minigene preparation) | APC.Ex7.InFus.BamHI-F | AAGAAGTGCAGGATCCGAACTGACCCCAATTTGTTATTAAAGG |
|  | APC.Ex7 InFus MluI-R | TCAAAACAAGACGCGTTGTAAACTGACAGCTAAAGTAAGGTATC |
| Sequencing of minigene inserts | pCAS-Seq-F | GGGTCAATAGCAGTGAGAGG |
|  | pCAS-Seq-R | GCTCCATTTCACAGGTAGAGA |
| RT-PCR and/or sequencing of RT-PCR products | pCAS-KO1-F | TGACGTCGCCGCCCATCAC |
|  | 6FAM-pCAS-KO1-F (5’-fluo) | TGACGTCGCCGCCCATCAC |
|  | pCAS-2R | ATTGGTTGTTGAGTTGGTTGTC |

Supporting Table 2. Primers used in the pCAS2 minigene splicing assay.
